# Research Progress of Maternal Metabolism on Cardiac Development and Function in Offspring

**DOI:** 10.3390/nu15153388

**Published:** 2023-07-30

**Authors:** Zhuoran Ren, Sisi Luo, Jiajun Cui, Yunhui Tang, Hefeng Huang, Guolian Ding

**Affiliations:** 1 Obstetrics and Gynecology Hospital, Institute of Reproduction and Development, Fudan University, Shanghai 200001, Chinahuanghefg@fudan.edu.cn (H.H.); 2 Research Units of Embryo Original Diseases, Chinese Academy of Medical Sciences, Shanghai 200032, China; 3 Shanghai First Maternity and Infant Hospital, Shanghai 200126, China

**Keywords:** DOHaD, metabolism, maternal diabetes, maternal obesity, cardiovascular diseases, congenital heart defects

## Abstract

The developmental origin of health and disease (DOHaD) hypothesis refers to the adverse effects of suboptimal developmental environments during embryonic and early fetal stages on the long-term health of offspring. Intrauterine metabolic perturbations can profoundly impact organogenesis in offspring, particularly affecting cardiac development and giving rise to potential structural and functional abnormalities. In this discussion, we contemplate the existing understanding regarding the impact of maternal metabolic disorders, such as obesity, diabetes, or undernutrition, on the developmental and functional aspects of the offspring’s heart. This influence has the potential to contribute to the susceptibility of offspring to cardiovascular health issues. Alteration in the nutritional milieu can influence mitochondrial function in the developing hearts of offspring, while also serving as signaling molecules that directly modulate gene expression. Moreover, metabolic disorders can exert influence on cardiac development-related genes epigenetically through DNA methylation, levels of histone modifications, microRNA expression, and other factors. However, the comprehensive understanding of the mechanistic underpinnings of these phenomena remains incomplete. Further investigations in this domain hold profound clinical significance, as they can contribute to the enhancement of public health and the prevention of cardiovascular diseases.

## 1. Introduction

The developmental origin of health and disease (DOHaD) hypothesis, first proposed by Baker in the late twentieth century, posits that early-life environmental insults, including those occurring during conception, gestation, and the perinatal period, can significantly impact an individual’s susceptibility to disease later in life [1]. The prevalence of maternal obesity and diabetes has risen dramatically over the past several decades and constitutes a major public health concern [2]. Maternal hyperglycemia has been linked to an elevated risk of numerous birth defects and long-term health issues in offspring. Several large-scale cohort studies have demonstrated that maternal metabolic status, including diabetes, obesity, and undernutrition, can impact both the morphological and functional development of offspring hearts, as well as their long-term cardiac health [3,4]. Despite these findings, further research is required to elucidate the precise effects of maternal metabolism on cardiac development and function in offspring, as well as the underlying molecular and cellular mechanisms involved.

The heart is one of the earliest organs to develop during embryonic development, with cardiogenesis beginning at approximately 15–19 days of human pregnancy and embryonic day 6.5–7.5 (E6.5–7.5) in mice. Initially, mesodermal cells form a heart-forming region known as the cardiac crescent, which contains myocardial precursor cells. These cells can be distinguished based on their temporal expression of various marker genes and are classified as either first heart field (FHF) or second heart field (SHF) cells. FHF cells ultimately become the heart tube, which then forms the definitive left ventricle and atrioventricular canal, while SHF cells give rise to the right ventricle, outflow tract, and atria. The primary heart tube, which is a looped tube with only one cavity, forms from around 20 to 25 days of human development and 9 days of mouse development. The heart tube then loops and twists to ensure proper cardiac segment alignment, in preparation for chamber septation. Atrial and ventricular septation begins at 31–35 days of human pregnancy and E10.5 in mice. By E13.5–14.5, the basic structure of the four chambers has formed [1,2]. After birth, cardiomyocytes retain some proliferative capacity, but this capability diminishes rapidly during the first week [3]. Therefore, precise regulation of gene expression is crucial for correct cardiac morphogenesis and differentiation, requiring the coordinated activity of many signaling pathways, such as HIF [4], Notch [5], and Hippo [6]. Any dysregulation of these pathways during embryonic development may disrupt the spatiotemporal regulation of complex three-dimensional heart structures, leading to morphological abnormalities, such as congenital heart diseases (CHDs), or cardiac dysfunction in offspring. The heart has a high energy demand to maintain a constant cardiac impulse, and thus its metabolic pattern is unique and worthy of attention. During the embryonic stage, the fetal heart primarily depends on glycolysis to acquire energy [7]. One unique characteristic of heart metabolism is that shortly after birth, it undergoes a dramatic transformation and primarily depends on fatty acid oxidation (FAO) to provide energy [8]. Some studies have suggested that adverse maternal environments may lead to early onset of this transformation and influence offspring’s cardiac metabolism and health [9].

Although a considerable body of evidence indicates that maternal nutritional status can influence fetal development, the specific effects on cardiac development and the underlying mechanisms remain poorly understood. Therefore, the objective of this review is to comprehensively assess the current state of research pertaining to maternal metabolism and its impact on cardiac development and function in offspring. By examining and synthesizing the existing literature, this review aims to provide an updated overview of the relationship between maternal nutritional status and cardiac outcomes in offspring, shedding light on the existing knowledge gaps and future research directions.

## 2. Methods

### 2.1. Search Strategy

We performed a systematic literature search on PubMed (1955 to 2023) and Web of Science Core Collection to identify relevant papers using the search strategy ‘maternal AND (hyperglycemia OR diabetes OR gestational diabetes OR pregestational diabetes OR Obesity OR BMI OR overweight OR undernutrition OR famine) AND (offspring OR neonatal OR birth OR fetal OR embryonic OR next generation) AND (heart OR cardiac OR cardiomyocytes OR cardiovascular)’. Additional articles were identified by reviewing reference lists of articles.

### 2.2. Inclusion/Exclusion Criteria

**Figure 1:**
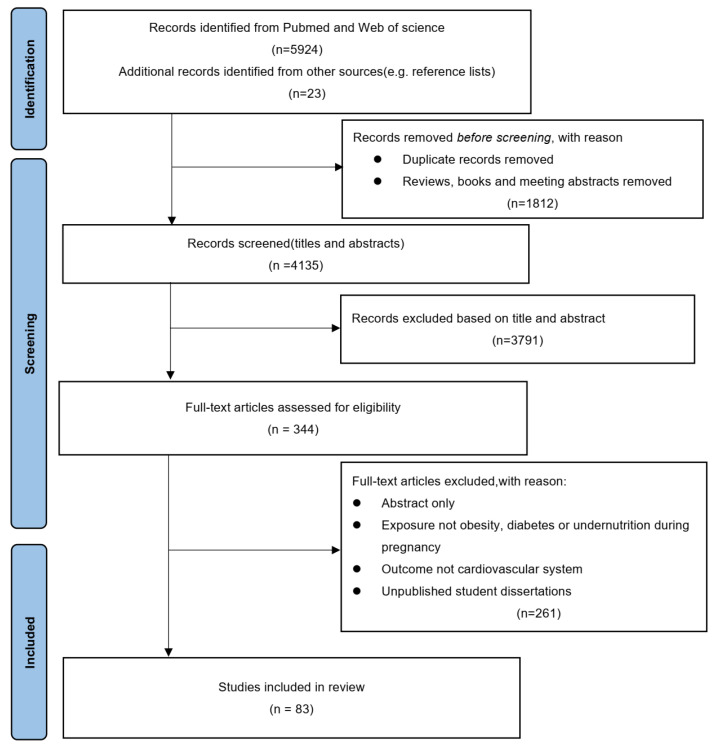
The methodology used for the literature search.

Studies were initially eligible if they met the following criteria: 1. Studies were published in English; 2. Studies were observational studies, randomized controlled trials, or observational studies were included. Reviews, meeting abstracts, and books were excluded. 3. The exposures of interest were maternal overweight, diabetes, and undernutrition. Diabetes includes type I diabetes mellitus, type II diabetes mellitus, and gestational diabetes mellitus. 4. The outcomes of interest was any kinds of cardiovascular diseases (CVDs) including CHDs, cardiometabolic risk factors, and diseases following ICD-8 and ICD-10 codes for CVDs, such as ischemic heart disease, cerebrovascular disease, stroke, heart failure, atrial fibrillation, hypertensive disease, deep vein thrombosis, pulmonary embolism, and other types of CVDs. Finally, 83 studies were included in this review (Figure 1).

## 3. Results

### 3.1. Human Studies of the Effect of Maternal Metabolism on Cardiac Development and Function in Offspring

#### 3.1.1. Maternal Diabetes

Exposure to hyperglycemia during early stages of life can have a profound impact on the cardiac health of offspring across their entire lifespan, ranging from the fetal stage to adulthood [10]. In fact, even during fetal development, the cardiac function of offspring can be adversely affected by hyperglycemia. Studies using fetal echocardiography have demonstrated that fetuses of diabetic mothers exhibit a range of morphological and functional changes across all stages of gestation, including septal hypertrophy and ventricular dysfunction [11,12].

Maternal diabetes is associated with an increased risk of birth defects, including CHDs, which are one of the most prevalent congenital anomalies and a common cause of perinatal mortality [13]. Extensive evidence has shown that both type 1 and type 2 diabetes enhance the risk of all subtypes of CHDs, such as septal and conotruncal defects [11,13,14,15,16,17,18]. For example, a cohort study following over 2 million births for 34 years showed that maternal pre-gestational diabetes mellitus (PGDM) was linked to a fourfold increase in offspring CHD incidence, with both types of diabetes showing similar elevated risks of CHDs. Moreover, mothers with acute diabetic complications conferred even higher increases, by up to eightfold, in CHD risk in offspring. Glucose regulation and insulin treatment does not reduce the odds of CHD in offspring of diabetic mothers [14]. Subclinical abnormalities in glucose levels are also related to an increased risk of CHDs. Priest et al. found that maternal glucose levels during the second trimester were strongly associated with the risk of tetralogy of Fallot compared with women who delivered infants without structural malformations, indicating that blood glucose level itself might be a continuous variable affecting offspring’s cardiac structure development [15]. Therefore, the prevention and treatment of maternal diabetes during pregnancy may play a critical role in reducing the incidence of CHDs in offspring.

Offspring born without CHDs can still exhibit altered cardiometabolic and functional phenotypes throughout their lifespan due to exposure to maternal hyperglycemia. A study reported that maternal hyperglycemia during the first half of pregnancy was associated with lower childhood left ventricular mass and left ventricular end-diastolic volume [5]. Offspring of mothers with gestational diabetes mellitus (GDM) exhibited higher blood pressure and cardiac structural changes during early childhood [19,20]. Kaseva et al. assessed cardiometabolic biomarkers and blood pressure in adult offspring of mothers with GDM and found increased markers of insulin resistance and a more atherogenic lipid profile [21]. Moreover, a cohort study indicated that intrauterine exposure to hyperglycemia was associated with higher mortality and an increased risk of cardiovascular diseases during early adulthood [22]. The longest cohort study to date, spanning 40 years, found that offspring of mothers with diabetes had an increased risk of early onset of various subtypes of cardiovascular diseases, such as hypertensive disease, heart failure, stroke, and ischemic heart disease [10]. Collectively, these findings suggest that intrauterine exposure to maternal diabetes can have lasting effects on the offspring’s cardiometabolic and functional health not only during the intrauterine stage but also throughout their lifespan.

#### 3.1.2. Maternal Obesity

Observational studies have consistently reported an association between maternal obesity during pregnancy and an increased risk of obesity, coronary heart disease, stroke, and other diseases in offspring [23]. Additionally, maternal obesity has been implicated in an increased incidence of CHDs in offspring. A population-based cohort study conducted in Sweden comprising 2,050,491 live singleton fetuses born between 1992 and 2012 found that the adjusted incidence of various CHDs, such as tetralogy of Fallot (ToF), transposition of the great arteries, and atrioventricular septal defects, was significantly higher in the obese group and correlated with higher maternal body mass index (BMI) [24]. Similarly, a systematic review of 24 studies revealed a dose-dependent relationship between maternal overweight, mild and severe obesity, and the incidence of all types of CHDs in offspring [25]. However, a Mendelian randomization study did not demonstrate a causal association between maternal BMI and the incidence of CHDs in offspring [26]. The conflicting results of these studies suggest that the effect of maternal obesity on the offspring’s heart remains uncertain, and additional robust evidence and analytical methods are necessary to control for potential confounding factors.

In a cohort study comprising 37,709 individuals, it was found that offspring of mothers with a BMI exceeding 30 exhibited elevated all-cause mortality compared to those born to mothers with normal BMI. Moreover, the offspring of obese mothers were at a higher risk of hospital admission for cardiovascular diseases in adulthood according to Persson et al. [27] Another study assessing neonatal cardiac structure and function found that newborns of obese mothers displayed an increased heart rate, reduced heart rate variability, and smaller left ventricular volume [28]. A follow-up echocardiography of infants born to overweight mothers at birth, three months, six months, and 12 months old, indicated that those born to overweight mothers, particularly in the third trimester, exhibited a thicker posterior left ventricular wall and elevated stroke volumes throughout the first year of life [29]. These early-life modifications could potentially suggest an increased risk of cardiovascular disease in adulthood.

#### 3.1.3. Maternal Undernutrition

Maternal undernutrition during gestation can have long-lasting effects on offspring health later in life [30]. Studies conducted on humans born around 1960 during the Chinese famine found that early-life exposure to famine, particularly during infancy, increased the risk of hypertension in adulthood [31,32]. Similarly, many studies have focused on the 1944–1945 Dutch famine and its effects on offspring health [33,34]. A historical cohort study conducted in Amsterdam, including 721 singleton men and women born during the Dutch famine, evaluated blood pressure response to stress and showed that gestational famine exposure was associated with a greater increase in blood pressure [35]. Two cohort studies that focused on the Dutch famine evaluated the prevalence of coronary heart diseases in offspring and suggested that maternal malnutrition may increase the occurrence of coronary heart diseases in adult offspring. However, given that both sample sizes were small, more evidence is needed to confirm this finding [36,37]. Researchers followed fetuses with growth restriction identified in fetal life and found that, during childhood (around the age of 5), children with fetal growth restriction exhibited a different cardiac shape and reduced stroke volume, which could explain their increased susceptibility to cardiovascular diseases in adult life [38]. Another study examined cardiac risk markers of adult men and women exposed to prenatal famine compared to their unexposed siblings. Their findings suggest that no increase was observed in the exposed group after adjusting for age and sex [39]. Ekamper et al. examined 41,096 men born around the time of the Dutch famine and evaluated the relationship between early life stage exposure to famine and mortality up to age 63 years. They found no increase in mortality from cardiovascular diseases compared to the control group [40]. Considering the challenges of acquiring human samples, more evidence and logical statistical methods are required to confirm the relationship between maternal malnutrition and offspring cardiac influence and eliminate confounding factors.

In the human studies above, it is evident that intrauterine exposure to metabolic disorders, including hyperglycemia, obesity, and maternal undernutrition, exerts long-term structural and functional influences on offspring’s cardiac health (Table 1). These findings underscore the significant impact of maternal metabolic disturbances during pregnancy on the developmental trajectory and functional integrity of the offspring’s cardiovascular system over an extended period.

### 3.2. Animal Models of the Effect of Maternal Metabolism on Cardiovascular Disease in Offspring

#### 3.2.1. Maternal Metabolism and the Risk of Congenital Heart Diseases in Offspring

Similar to findings from human studies, animal studies have also demonstrated the detrimental effects of maternal metabolic disorders on fetal heart development, leading to a significantly increased risk of CHDs. In rodent models, diabetes or obesity can be induced by streptozocin injection or a high-fat diet to mimic the overnutrition state in humans. Single-cell transcriptomic profiling of embryonic hearts showed that exposure to hyperglycemia environment as early as E9.5 and E11.5 led to diverse cellular responses, with differentially expressed genes (DEGs) enriched in pathways related to cell differentiation, voltage-gated calcium channels, potassium channels, regulators of cardiac contractility, and transcriptional and chromatin regulators. These changes were strongly perturbed in genes related to cardiomyocyte lineage and may be related to the spectrum of conotruncal defects observed in hyperglycemia-exposed fetuses [41].

Studies have also shown that diabetic pregnancies have deleterious effects on whole heart development, leading to various cardiac malformations, including atrial septal defect (ASD), ventricular septal defect (VSD), atrioventricular septal defect (AVSD), transposition of great arteries (TGA), double outlet right ventricle (DORV), and TOF [42,43,44,45,46,47,48,49,50,51,52,53,54,55]. At E13.5, when the four-chamber structure is normally formed, offspring of diabetic mothers displayed VSD, persistent truncus arteriosus (PTA), outflow tract defects, and disarranged myocardium, indicating congenital defects or delayed cardiac formation [46,55]. Prenatal and neonatal heart analysis revealed a spectrum of CHDs in offspring of diabetic mothers, with the majority of them being VSD [47,49,50], which is consistent with human cohort studies [14]. Additionally, even those without congenital morphological malformations, newborns of diabetic mice showed diminished systolic and diastolic function, with a significantly lower mean heart rate, shortening fraction, and E:A ratio [56].

Animal studies have also established maternal obesity models to investigate the influence of maternal body weight on offspring’s heart structure and function. Maternal obesity led to disrupted Ca^2+^ homeostasis and contractile dysfunction in fetal cardiomyocytes [57]. In miniature pig models, high-fat diets during gestation resulted in larger ventricular mass and volume, glycogen accumulation, and low oxidase activity at birth [29]. In mice offspring of mothers exposed to a high-fat diet during pregnancy, there was an increase in absolute heart weight, left ventricular wall, and septal thickness, and signs of impaired cardiac function such as decreased ejection fraction in adulthood (8 weeks of age) [58].

Maternal undernutrition also interferes with fetal heart development. To compensate for the lack of evidence from human studies, scientists have created a number of animal models to simulate the nutritionally restricted environment of early development. A low protein diet in dams before embryo implantation leads to early blastocysts of offspring exhibiting slow cell growth and hypertension at twelve weeks [59]. Elevated expression of IGF2/IGF2R signaling associated with hypertrophy in the fetal right ventricle was found to cause ventricular remodeling using a model of nutritional restriction during pregnancy in sheep [60]. Cardiomyocytes of intrauterine growth restriction (IUGR) fetuses exhibited a smaller size, less maturity, and reduced activity in the cell cycle [61]. The primate baboon model demonstrates that the left ventricular mitochondria of male zygotes are affected, as evidenced by increased mtDNA content, reduced mitochondrial number, and disrupted internal structure, which may affect the energy metabolism of the fetal heart [62].

#### 3.2.2. Maternal Metabolism and Long-Term Cardiac Diseases in Offspring

Offspring of diabetic mothers who do not exhibit morphological abnormalities may still suffer from impaired cardiac function throughout their lives (Table 2). Newborns of diabetic mothers have been shown to have lower birth weight, cardiomyopathy, and diminished systolic and diastolic function. Although their cardiac function improves after birth and into adulthood, poorer cardiac function re-emerges in aged offspring, particularly in male offspring [63]. Hypertension has been observed in male offspring of mothers with diabetes at the age of 6 months [64]. While many studies have demonstrated that cardiac function in adult offspring is not obviously influenced under baseline conditions, it is more susceptible to environmental stimulation or cardiovascular diseases. For example, one study found that before dietary challenges, echocardiography showed no significant alterations comparing the diabetic adult group and the control group. However, after 28 weeks of a high-fat diet, the diabetic group exhibited cardiomyocyte hypertrophy, increased inflammation reaction, and cardiovascular risks, while the control group showed no such changes [65]. Offspring exposed to intrauterine hyperglycemia also showed a diminished tolerance to myocardial ischemia. Our previous research found that after myocardial ischemia insult and reperfusion, male offspring of the diabetic group had a larger infarct size and aggravated cell apoptosis [66]. Consistent with our study, Chen et al. demonstrated that after 24 h and seven days of ischemic insult, male offspring showed greater susceptibility and cardiac dysfunction than the control group [67]. Nevertheless, the mechanism of the persistent effects of maternal hyperglycemia is still not fully understood.

Another study revealed that adult offspring of obese mice showed left ventricular diastolic dysfunction that worsened progressively in females, but not in males [68]. Offspring exposed to maternal obesity exhibited mild cardiac dysfunction, which further worsened under hypertension stress, leading to severe cardiac remodeling and malfunction [69]. Maternal high-fat diet induced cardiac hypertrophy only in male offspring, but not in females. Both sexes showed no impairment in systolic and diastolic function, but male offspring exhibited increased susceptibility to ischemia-reperfusion injury in adulthood [70]. Male offspring of mothers on a low-protein diet exhibited elevated systolic blood pressure at 9 and 15 weeks of age, and at 21 weeks of age for both sexes [71].

### 3.3. Mechanisms of the Effect of Maternal Metabolism on Cardiac Development and Function in Offspring

#### 3.3.1. Maternal Metabolism Influences Cardiac Mitochondria in Offspring

Cardiomyocytes from neonatal rats born to diabetic mothers exhibited reduced mitochondrial function, lower mitochondrial DNA copy number, and abnormal structure and membrane potential [56,72]. Transcriptomics analysis revealed changes in the expression of several mitochondrial-specific genes due to maternal hyperglycemia and high-fat diet exposure [73]. Raji and colleagues monitored the cardiac health of offspring over an extended period and reported that male adult offspring from diabetic pregnancies showed decreased mitochondrial respiration and increased autophagy despite no significant differences observed in the weaning stage (21 days) [74]. Another study investigated cardiomyocyte activity from birth to old age, showing lower mitochondrial capacity in the diabetic group at birth, which improved after birth and showed no differences at 10 weeks. However, mitochondrial respiratory activity decreased again in aged (12-month-old) offspring, preceding cardiac dysfunction, which suggests that mitochondria may play a crucial role in enhancing susceptibility to cardiovascular insults in adult offspring [63].

#### 3.3.2. Nutritional Molecular Signals Can Directly Affect Cardiac Gene Expression

Glucose can act as a signaling molecule that directly interacts with RNA binding proteins to modulate gene expression and tissue differentiation [75]. The expression of GAB1, a gene involved in PI3K/Akt signaling, was found to be reduced in the fetal heart tissue of diabetic rats, contributing to an increased risk of congenital heart defects [76]. Additionally, genes associated with cardiac lipid metabolism, such as PPARα and PGC-1, were downregulated in the fetal and neonatal hearts of diabetic dams, potentially affecting heart energy utilization and cardiomyocyte cell cycle regulation [77,78]. Nutritional disturbances during intrauterine development can also lead to cardiac dysfunction, potentially via dysregulated AMPK/PPARα signaling and impaired vascular endothelial function in offspring of rats with gestational diabetes [79].

Transcriptional analysis of fetuses exposed to maternal obesity has identified alterations in the expression of several metabolic genes, including Pparg and Cd36, which are involved in lipid metabolism. In male fetuses, there was an increase in lipid synthesis and metabolism of membrane lipid derivatives, while in female fetuses, there was an enhancement in the absorption of monosaccharides and carbohydrates [68]. In cardiac progenitors of offspring from obese mothers, genes associated with extracellular matrix remodeling, metabolism, and TGF-β signaling were found to be dysregulated. Moreover, the expression of Nkx2-5, a crucial regulator of heart development, was correlated with maternal obesity in fetal mice and persisted in adult hearts [69]. Male offspring of obese mothers also showed increased cardiac angiotensin II receptor type 2 (Agtr2) mRNA and protein abundance, which might contribute to heightened cardiac ischemic vulnerability [70].

Maternal malnutrition can have significant effects on the nutrition usage and gene expression of offspring’s hearts. A low-protein diet during pregnancy may lead to compensatory increased metabolism during fetal and perinatal periods, mediated through adjustments in endocytosis during early fetal stages [80]. To compensate for the shortage of nutrients in the intrauterine environment, the offspring’s visceral nutritional transport cavity undergoes alterations, which might increase their susceptibility to cardiovascular diseases later in life [81]. Researchers have also found that males with IUGR show decreased expression of AMPK and ACC, which are crucial for fatty acid activation in the sarcoplasm and their transport into the mitochondria [82].

#### 3.3.3. Reactive Oxygen Species Could Mediate Modulating Reactions towards Environmental Changes

Reactive oxygen species (ROS) are produced as byproducts of normal mitochondrial metabolism and homeostasis, which include oxygen free radicals, such as the superoxide anion radical and the hydroxyl radical, as well as non-radical oxidants such as hydrogen peroxide [83]. The accumulation of potentially toxic levels of ROS and oxidative stress can have a detrimental effect on fetal development [84]. Metabolic disorders such as hyperglycemia can increase ROS production through various mechanisms, including increased polyol pathway flux, intracellular advanced glycation end products (AGEs), hexosamine pathway flux, and protein kinase C activation [85]. Studies have shown a significant increase in superoxide levels in the embryonic and neonatal hearts of diabetic dams compared to the control group [47,56], and these levels remained elevated during the adolescent period [67].

ROS can impact the cardiac health of offspring through various biological pathways. In adult offspring exposed to intrauterine hyperglycemia, an increased susceptibility to hypertension associated with arterial dysfunction has been observed. This effect is attributed to the inhibition of the nitric oxide (NO) pathway caused by elevated ROS levels [64]. The reduction of NO induced by hyperglycemia leads to an upregulation of *Jarid2*, an epigenetic repressor of *Notch1*. Consequently, diminished expression of *Notch1* below the threshold required for normal cardiac development increases the risk of CHD in the offspring of diabetic mothers [46].

Furthermore, downregulation of sirtuin 1 (Sirt1), a protective factor against cardiovascular diseases such as myocardial ischemia, has been observed in adult male offspring of mothers with GDM. This downregulation promotes myocardial mitochondrial autophagy, thereby contributing to the susceptibility of these individuals to myocardial ischemia. It was found that antioxidant treatment can restore Sirt1 repression and rescue the cardiac ischemia-sensitive phenotype induced by GDM [67].

Numerous studies have shown that antioxidants have the potential to decrease the incidence of CHDs and enhance cardiac health in offspring. For instance, administering tetrahydrobiopterin, a cofactor of endothelial NO synthase, to diabetic mice led to a significant reduction in CHD incidence, dropping from 59% to 27%, and prevented major abnormalities [45]. Similarly, *N*-acetylcysteine, which promotes glutathione synthesis and curbs ROS production, was able to decrease the occurrence of CHDs in the offspring of pregestational diabetic mothers [50]. Furthermore, supplementation of zinc during gestation demonstrates effective prevention of CHDs induced by maternal diabetes. This beneficial effect can be attributed to the ability of zinc to reduce lipid peroxidation, superoxide ions, and oxidized glutathione levels in the developing heart [53]. These findings strongly suggest that ROS reduction represents a promising therapeutic avenue for improving the cardiac health of offspring born to diabetic mothers.

#### 3.3.4. Epigenetic Regulation Plays a Vital Role in Cardiac Changes Induced by Maternal Nutritional Disorders

Epigenetics refers to the heritable changes in gene expression that occur without altering the underlying DNA sequence. There are three common forms of epigenetic regulation: DNA methylation, histone modification, and non-coding RNA [86]. Epigenetic regulation of cardiac genes holds significant importance in cardiac development [87]. Moreover, maternal nutritional dysfunction can exert a profound influence on offspring health through the mediation of epigenetic mechanisms [88].

DNA methylation. DNA methylation is a well-studied epigenetic mechanism during fetal development. Recent human studies have demonstrated that genome-wide DNA methylation changes occur in umbilical cord blood from diabetic mothers [89,90]. In addition, the global DNA methylation level of the placenta and peripheral blood was increased in children of mothers with GDM, with 48 differentially methylated CpG sites being identified [91,92]. Chen et al. found that the DNA methylation level was significantly increased in 6-week-old male offspring of diabetic mice, leading to decreased expression of Sirt1 and increased susceptibility to myocardial ischemia [67]. Furthermore, cardiomyocytes exposed to a hyperglycemic environment displayed altered chromatin accessibility, as determined by ATAC-seq profiling [46]. Analysis of DNA methylation in whole blood after exposure to prenatal malnutrition during the Dutch Famine revealed differentially methylated regions (DMRs) that mainly occurred at regulatory regions and mapped to genes enriched for differential expression during early development. These genes, such as CDH23, CMAD7, INSR, KLF13, CPT1A, and RFTN1, are involved in many metabolic and growth pathways [93]. When evaluating the DNA methylation level of adult sheep muscle tissue after periconceptional undernutrition, scientists identified 686 DMRs affecting genes related to the development and function of the muscular system and steroid hormone receptor activity, suggesting that maternal nutritional status may induce long-lasting epigenetic changes in the offspring, leading to cardiac dysfunction [93].

Histone modification. Histone modification is another common form of epigenetic change that regulates gene expression through post-translational modifications of histone proteins, such as methylation, sumoylation, phosphorylation, and acetylation [94]. By measuring histone marks and global DNA methylation levels, Blin et al. found that maternal exposure to a high-fat diet induced long-term derepressive chromatin marks in the adult offspring heart. Evidence suggests that maternal high-fat exposure upregulates cardiac developing genes, such as isl lim homeobox 1 (Isl1) and six homeobox 1 (Six1), by decreasing di- and trimethylated histone H3 and ubiquitinated histone H2A levels [95]. Chromatin immunoprecipitation sequencing studies have shown that the offspring of obese mothers exhibit a differential peak distribution on gene promoters related to the acetylation of lysine 9 and 14 and the trimethylation of lysine 4 and 27 in histone H3. Many of these genes are associated with metabolic processes and cardiac disease susceptibility [96].

MicroRNA and other non-coding RNA. MicroRNA and other non-coding RNA play a role in cardiovascular disease development [97]. The expression of microRNA is influenced by adverse intrauterine environments, which can affect fetal heart development [98]. MicroRNA profiling showed that in the offspring of pregestational diabetic mothers, 149 mapped microRNAs were altered, affecting 2111 potential microRNA target genes associated with cardiac development, such as STAT3 and IGF-1 and transcription factors Cited2, Zeb2, Mef2c, Smad4, and Ets1 [48]. In rats exposed to maternal high-fat diets, a downregulated subset of microRNAs was identified, many of which were related to FGFβ expression, suggesting that altered microRNA expression might be related to TGFβ-mediated cardiac fibrosis and hypertrophy induced by early exposure to an altered intrauterine environment [99]. In the heart tissue of young offspring exposed to maternal obesity, miR-133 was up-regulated, simulating the MAPK pathways and leading to cardiac hypertrophy at an early age [100]. Altered microRNA expression levels might explain transgenerational epigenetic changes in another way [98]. In a baboon model of moderate maternal undernutrition, 56 cardiac miRNAs were dysregulated in female offspring and 38 in male offspring [101]. Although other non-coding RNA such as long non-coding RNA, siRNA, and piRNA are also related to heart development [102], no evidence has linked them with metabolic disorders, requiring further investigations.

## 4. Conclusions and Future Directions

**Figure 2:**
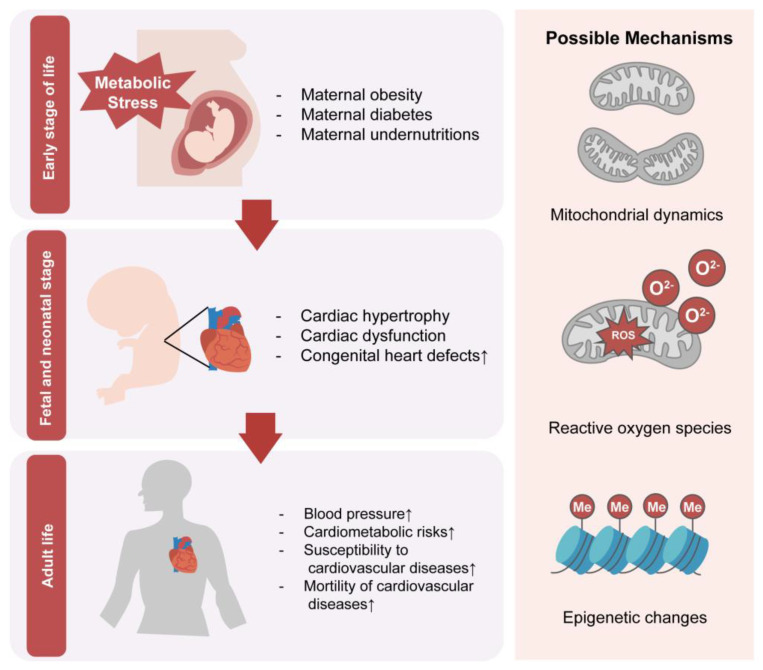
Maternal metabolism and cardiovascular diseases in offspring. Intrauterine exposure to nutritional disorders could influence offspring’s health through all stages of life. Possible mechanisms including mitochondrial changes, increase of reactive oxygen species, and epigenetic changes such as DNA methylation and histone modification.

Alterations in the intrauterine environment and nutrient availability can have significant impacts on offspring growth, development, and adult health. Changes in the accessibility of glucose, lipids, and proteins during fetal growth can affect cardiac development and increase the risk of long-term cardiovascular disease (Figure 2). However, the precise effects and underlying mechanisms of these relationships remain unclear due to limitations in human studies. Additional research is necessary to elucidate the pathways through which nutritional molecules influence fetal development, persist into adulthood, and contribute to distant morbidity and mortality. This would enable development of novel biomarkers and possible new intervention strategies. The identification of epigenetic markers as possible biomarkers has instructive effects on changing maternal lifestyles and possible supplements or diets, and blocking pathways related to cardiovascular disease in early life has significant implications for improving public health and preventing cardiovascular disease.

## Figures and Tables

**Table 1:** Human studies of the effect of maternal metabolism on cardiac development and function in offspring.

Study	Study Population	Exposures	Following Years	Outcomes	Major Findings
Øyen et al. [14]	2,025,727 persons born alive in Denmark between 1978 and 2011	PGDM GDM	Birth defects	All types of congenital heart diseases	CHD risk increased nor differed by diabetes subtype Diabetes complication related with higher risk
Priest et al. [15]	277 pregnant women in southern and central California	Maternal diabetes	Birth defects	Cardiac malformations, including ToF, d-transposition of the great arteries (dTGA)	Odds of ToF increased dTGA not changed
Tam et al. [19]	970 mothers who joined the Hyperglycemia and Adverse Pregnancy Outcome study and their children	GDM	7 years	Cardiometabolic risk	Associated with offspring’s risk of abnormal glucose tolerance, obesity, and higher blood pressure Sex dimorphism, apparent in girls
Kaseva et al. [21]	906 pregnant women and their offspring from Uusimaa and northern Finland	GDM Obesity	24.1 ± 1.3 years	Cardiometabolic risk	Increased markers of insulin resistance in GDM offspring Impaired glucose regulation in obesity offspring
Guillemette et al. [22]	293,546 people born between 1979 and 2005 in Manitoba, Canada	PGDM (type II) GDM	Up to 35 years	Cardiovascular disease, including cardiac arrest, myocardial infarction, ischemic heart disease, and cerebral infarction	Higher morbidity and higher risk of cardiovascular disease
Yu et al. [10]	All 2,432,000 liveborn children without congenital heart disease in Denmark during 1977–2016	PGDM (including type I and type II) GDM	40 years	Early onset CVD including ischemic heart disease, cerebrovascular disease, stroke, heart failure, atrial fibrillation, hypertensive disease, deep vein thrombosis, pulmonary embolism, other CVDs	Increased rates of early onset CVD from childhood to early adulthood
Persson et al. [24]	2,050,491 live singleton infants born between 1992 and 2012 in Sweden	Overweight/obesity	Birth defects	Congenital heart defect	Aortic branch defects, ASD, and persistent ductus arteriosus increase
Groves et al. [28]	87 neonates in UK	Obesity	Newborns	Heart rates Heart variability Cardiac function	Increased heart rate Decreased heart rate variability Decreased left ventricular volumes
Guzzardi et al. [29]	91 pregnant women in Italy	Overweight	12 months	Cardiac function	Cardiac morphology changed
Wang et al. [31]	1966 adults born between 1956 and 1964 in China	Maternal malnutrition	45 years	Hypertension	Increased risk of hypertension
Painter et al. [35]	721 men and women born as term singletons in Amsterdam at about the time of the Dutch 1944–1945 famine	Maternal malnutrition	58 years	Hypertension	Increased blood pressure in famine exposed individuals
Roseboom et al. [36]	912 singletons born in Amsterdam at about the time of the Dutch 1944–1945 famine	Maternal malnutrition	50 years	Coronary artery disease (CAD)	Increased risk of coronary heart diseases
Painter et al. [37]	837 singletons born in Amsterdam at about the time of the Dutch 1944–1945 famine	Maternal malnutrition	50 years	CAD	Increased risk of coronary heart disease
Lumey et al. [39]	1075 men and women born around the Dutch 1944–1945	Maternal malnutrition	58 years	CAD	No relation between prenatal famine and CAD
Ekamper et al. [40]	41,096 men born in 1944–1947 in the Netherlands	Maternal malnutrition	63 years	Mortality of heart diseases	No increase in mortality from cardiovascular diseases

**Table 2:** Long-term cardiac effects of offspring exposed to maternal overnutrition in early stage of life.

Study	Species	Gender	Age	Early Exposure	Second Hit	Major Findings
Pereia et al. [63]	Rat (Sprague Dawley)	M	P1, 3 W, 10 W, 6 W, 12 W	HF group: Gestational diabetes group:	-	Declined cardiac function in aged offspring
Louwagie et al. [64]	Rat (Sprague Dawley)	M	12 W, 16 W, 20 W, 24 W	Streptozotocin, 35 mg/kg, IP, at E0	-	Higher blood pressure
Yu et al. [65]	Rat (Sprague Dawley)	M	8 W, 29 W–36 W	Transgenic Tet29 female dams, 1.5 mg/kg DOX in drinking water	High-fat diet challenge for 28 weeks	Cardiac dysfunction LV hypertrophy Altered proinflammatory status
Schütte et al. [66]	Mouse (C57BL/6)	M	8–10 W	STZ, IP, 80 mg/kg for 3 days at 8 weeks old	Myocardial ischemia/reperfusion injury at 8–10 weeks old	Larger infarct size Augmented cardiac dysfunction Augmented myocardial apoptosis
Gao et al. [67]	Rat (Sprague Dawley)	M and F	6 W	STZ, SC, 50 mg/kg at E12	Heart ischemia for 24 h at 6 weeks-old	Larger infarct size LV dysfunction in male offspring
Chen et al. [68]	Mouse (C57BL/6)	M and F	E18.5, 3 W, 6 W, 9 W, 24 W	Obesogenic diet until body weight increased for 25%	-	Altered embryonic metabolic genes transcription Diminished cardiac diastolic function Female cardiac function worsens with age
Vaughan et al. [69]	Mouse (Chimeric)	M	E18.5, 16 W, 32 W	Obesogenic diet from 4 weeks old through whole life	Isoproterenol (60 mg/kg/day) in 8-week-old mice for 14 days	Exacerbated cardiac remodeling Altered gene expression of cardiac progenitors
Ahmed et al. [70]	Rat (Sprague Dawley)	M and F	3 W	High-fat diet from E0 to E21	Myocardial ischemia/reperfusion injury	Cardiac hypertrophy in male Increased infract size in male

M, Male; F, Female; P, postnatal day; W, weeks; E, embryonic day; M, month; lv, left ventricular.

## Data Availability

No new data were created.
